# Influence of hearing in the non-implanted ear and age on long-term speech recognition benefit for adult cochlear implant users with asymmetric hearing loss

**DOI:** 10.3389/fnins.2026.1871719

**Published:** 2026-09-01

**Authors:** Sylvia Mihailescu, Samantha P. Scharf, Nicholas J. Thompson, Margaret E. Richter, Matthew M. Dedmon, Amanda D. Sloop, Kevin D. Brown, Margaret T. Dillon

**Affiliations:** 1 Department of Otolaryngology-Head and Neck Surgery, University of North Carolina at Chapel Hill, Chapel Hill, NC, United States; 2 School of Medicine, University of North Carolina at Chapel Hill, Chapel Hill, NC, United States

**Keywords:** bimodal, cochlear implant, single-sided deafness, spatial hearing, spatial release from masking (SRM), unilateral hearing loss (UHL)

## Abstract

Most cochlear implant (CI) users with normal hearing or mild to moderately-severe hearing loss in the non-implanted ear, known as asymmetric hearing loss (AHL), experience significant benefits in spatial hearing as compared to pre-operative abilities, though individual outcomes vary. This study analyzed the influence of the hearing in the non-implanted ear and age on long-term outcomes for 35 adult CI users with AHL. Unaided thresholds were measured behaviorally at annual post-activation visits and were used to calculate a pure-tone average (PTA; 0.5, 1, 2, 4 kHz). Sentence recognition in noise was assessed with the AzBio sentences in a 10-talker masker with the target presented from the front loudspeaker and the masker presented 90° toward the implanted ear, 90° towards the non-implanted ear, or co-located with the target. Spatial release from masking was calculated as the benefit in speech recognition when the masker was presented towards the implanted ear (SRMci) or the non-implanted ear (SRMcontra) as compared to the co-located configuration. Spatial release from masking did not change significantly after 1-year post-activation for SRMci and continued to significantly improve for SRMcontra. Hearing in the non-implanted ear significantly influenced SRMci. Age at surgery significantly influenced SRMcontra. Six (17%) participants experienced a PTA decline of ≥ 15 dB HL in the non-implanted ear, which negatively influenced spatial hearing. For those, performance generally improved with adjustments to the hearing aid fitting or after bilateral cochlear implantation. Long-term outcomes for adult CI users with AHL are significantly influenced by hearing in the non-implanted ear and age – though differentially across speech-in-noise configurations.

## Introduction

Candidacy criteria for cochlear implantation have expanded to include individuals with asymmetric hearing loss (AHL), defined as profound hearing loss in the ear-to-be implanted and either normal hearing (e.g., single-sided deafness) or mild to moderately-severe hearing loss in the contralateral ear (US FDA, 2019a, b). Most adult cochlear implant (CI) users with AHL experience benefits in the bilateral condition within the initial months following CI activation as compared to pre-operative abilities or in an unaided (monaural) listening condition (Arndt et al., 2011; Buss et al., 2018; Deep et al., 2021; Dillon et al., 2020, 2022, 2026; Firszt et al., 2012, 2018, 2023; Gartrell et al., 2014; Johnson et al., 2025; Peters et al., 2021; Vermeire and Van de Heyning, 2009). One method to assess the benefit of listening with both ears is to compare the speech recognition in noise when the target and masker are co-located versus spatially-separated. The benefit observed when the masker is spatially-separated from the target on the horizontal plane as compared to co-located is known as spatial release from masking (SRM; Bronkhorst and Plomp, 1988; Freyman et al., 2001; Gallun et al., 2005). For adult CI users with AHL, some data suggest that individual variability in SRM may be influenced in part by hearing in the non-implanted ear and/or age at surgery (Dillon et al., 2020; Mertens et al., 2017; Thompson et al., 2023). What remains relatively unknown is the influence of these factors on the long-term outcomes for adult CI users with AHL.

Individual outcomes for adult CI users with AHL have been shown to be influenced in part by the unaided thresholds in the non-implanted ear, with better outcomes for those with better acoustic hearing (Dillon et al., 2026; Firszt et al., 2018; Johnson et al., 2025; Mertens et al., 2017). For example, Dillon et al. (2020) conducted an exploratory review of the influence of hearing in the non-implanted ear on SRM for 35 adult CI users with AHL at 1-year post-activation. They observed that better hearing in the non-implanted ear may be associated with greater SRM when the masker was spatially-separated towards the implanted ear as compared to co-located (SRMci); however, this was not disentangled from the potential influence of age, which was significantly correlated with the hearing in the non-implanted ear. There is a need to assess SRM after long-term device use (e.g., >1 year post-activation) for CI users with AHL and analyze the potential influence of hearing in the non-implanted ear on SRM.

A consideration when analyzing the influence of the hearing in the non-implanted ear on outcomes, such as SRM for CI users with AHL, is whether that hearing significantly changes over the years following device activation. The incidence of significant changes in unaided thresholds in the non-implanted ear for this patient population is not well understood. Speck et al. (2024) reviewed the audiograms of 323 individuals with AHL over time and found that 104 (32%) individuals experienced a decline in the pure-tone average (PTA; 0.5, 1, 2, & 4 kHz) in the better hearing ear from a mean of 21 dB HL (SD: 12 dB HL) to a mean of 36 dB HL (SD: 20 dB HL). For that sub-sample, the decline in hearing occurred at an average of 5 years (SD: 6 years) from the time of diagnosis. Due to the potential impact of hearing in the non-implanted ear on outcomes for CI users with AHL, implementation of clinical protocols that routinely monitor unaided thresholds and timely intervention (e.g., new hearing technology) when needed may be critical.

The SRM for CI users with AHL may also be influenced by the individual’s age. Older adults tend to experience less SRM than younger adults and require larger separations between the target and masker before benefiting from the spatial separation (Gallun et al., 2013; Srinivasan et al., 2016). Also, decline in processing speed (Salthouse, 2000) and temporal processing (Grose et al., 2006; Grose and Mamo, 2010) can occur with aging, which could negatively influence performance on measures of speech recognition in noise. Considering these findings, variability in individual outcomes on measures of SRM may be due in part to aging.

The present study analyzed the influence of hearing in the non-implanted ear and age on the long-term SRM for CI users with AHL. Unaided thresholds in the non-implanted ear were assessed at annual post-activation visits to analyze whether hearing for the group changed significantly over time. We hypothesized that SRM would be significantly influenced by hearing in the non-implanted ear and age. For those with a change in the PTA in the non-implanted ear of ≥ 15 dB HL, individual data were reviewed to calculate the incidence of substantial changes in unaided thresholds and to review their performance before and after modifications to their current technology or fitting of new hearing technology (e.g., second side CI).

## Methods

A repeated-measures investigation of unaided thresholds and aided speech recognition was conducted over time for adult CI users with AHL. Study procedures were approved by the study site Institutional Review Board (IRB# 10-0473) and participants provided written consent. Participants had originally received their CI as part of a clinical trial investigating the effectiveness of CI use for individuals with AHL (see Buss et al., 2018; Dillon et al., 2020). The clinical trial had recruited two groups of participants. The first group (*n* = 20) were participants with a moderate-to-profound sensorineural hearing loss (SNHL) in one ear and normal or near-normal hearing (defined as thresholds ≤ 35 dB HL from 125 to 8,000 Hz) in the other ear (see Buss et al., 2018). After maximum enrollment in the first group, recruitment initiated for the second group (*n* = 20) of participants who had an unaided pure tone average (0.5, 1, & 2 kHz) of ≥ 70 dB HL in one ear and between 35 and 55 dB HL in the other ear (see Dillon et al., 2020). For both groups, pre-operative aided word recognition was ≤ 60% in the ear-to-be implanted and ≥ 80% in the contralateral ear. Potential candidates were excluded if they reported tinnitus to be severe or catastrophic on the Tinnitus Handicap Inventory (Newman et al., 1996), and/or if there was evidence of a conductive hearing loss, cochlear ossification, or retrocochlear pathology. That clinical trial’s endpoint for each participant was the 1-year post-activation visit. For the present study, participants from the original clinical trial were recruited and assessed annually in combination with their routine clinical follow-up visits.

Measures of unaided audiometric thresholds and aided speech recognition in noise were completed with the participant seated in a single- or double-walled soundbooth. Unaided thresholds were measured behaviorally using pulsed pure tones presented via insert earphones. The PTA for the non-implanted ear was calculated for each visit. Aided speech recognition was assessed with the AzBio sentences (Spahr et al., 2012) in a 10-talker masker. The participant was seated in the center of a 180° loudspeaker arc, with each loudspeaker ~1 meter away. The target was presented at 0° azimuth (S0). The masker was either co-located with the target (S0N0), presented 90° towards in the implanted ear (S0Nci), or presented 90° toward the non-implanted ear (S0Ncontra). The signal-to-noise ratio (SNR) was either 10, 5, or 0 dB. The specific SNR was originally determined at the 1-month post-activation interval during the clinical trial as the SNR that the participant scored ≤ 50% correct in the S0N0 configuration (see Buss et al., 2018). Participants were instructed to repeat the target sentence and respond with their best guess when unsure. Performance was scored as the percent of words correctly repeated for each 20-sentence list (one list per target-to-masker configuration). The score in the S0N0 configuration was subtracted from each spatially-separated configuration (i.e., S0Nci and S0Ncontra) to calculate the SRM (SRMci and SRMcontra, respectively). Participants listened with their familiar, every-day CI map. Participants with a mild-to-moderate hearing loss in the non-implanted ear listened with a hearing aid; the output of the hearing aid was verified against NAL-NL1 prescriptive targets (Byrne et al., 2001) prior to assessment. Participants with a mild-to-moderate hearing loss in the non-implanted ear who did not use a hearing aid (see below) were fit with a hearing aid for the aided assessments.

### Data analysis

A linear mixed effects (LME) model analyzed the main effects and interaction of visit (pre-operative and annual post-activation visits; continuous variable) and age at surgery (years; continuous variable) on the PTA in the non-implanted ear at each visit (dB HL; continuous variable). For long-term SRM, LME models analyzed the main effects of annual follow-up visit (years since CI activation), age at surgery, and PTA in the non-implanted ear on SRMci and SRMcontra. Analyses were conducted using the R software (R Core Team, 2020) with participant included as a random factor. Significance was defined *p* < 0.05.

Individual-level data were also reviewed for those whose PTA in the non-implanted ear declined by ≥ 15 dB HL. This criterion was selected since a ≤ 10 dB HL threshold change is clinically considered for test–retest reliability (American National Standards Institute S3.21-2004) and that a PTA decline of ≥15 dB HL was a conservative estimate to capture individuals who would need a change in their device fitting or recommended hearing technology.

## Results

Thirty-five participants (19 female) from the original clinical trial sample (*n* = 40) enrolled in the present study and provided long-term data (≥ 2 years post-activation). The etiology of the hearing loss in the implanted ear was reportedly: Meniere’s disease (*n* = 5), noise induced hearing loss (*n* = 1), viral infection (*n* = 1), trauma (*n* = 1), or unknown (*n* = 27). Most cases were sudden in onset (*n* = 24). The reported duration of moderate-to-profound SNHL in the implanted ear prior to cochlear implantation ranged from 2 months to 9.7 years (mean: 3.1 years; SD: 2.3 years). The mean age at CI surgery was 59 years (SD: 14 years), with a range of 23 to 77 years. Participants were implanted with a MED-EL 12-channel Standard straight electrode array (31.5 mm). At the time of data review, the most recently completed post-activation visit ranged from 2.1 to 10.2 years (mean: 7.0 years; SD: 2.5 years).

**Figure 1:**
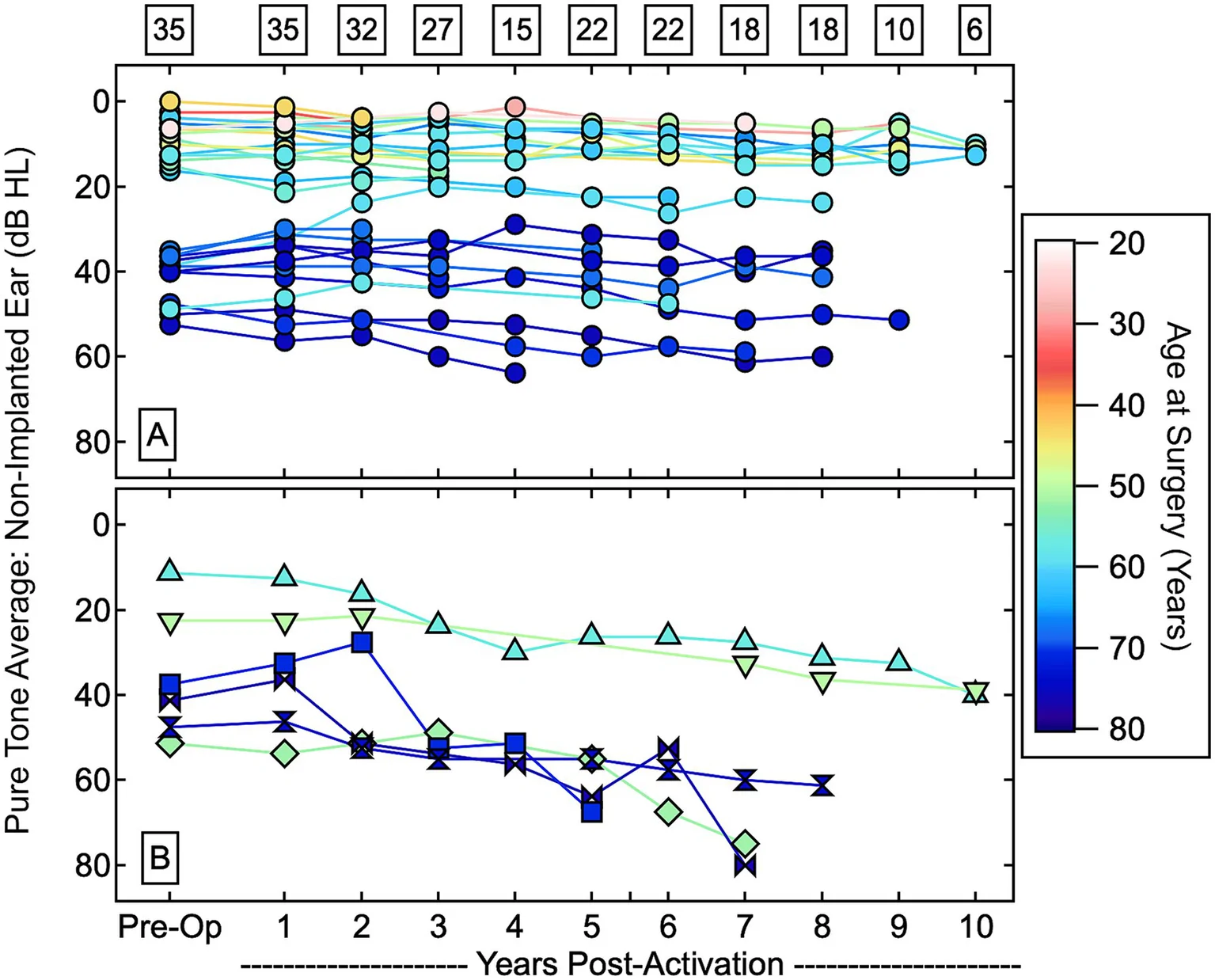
Unaided pure tone average (PTA: 0.5, 1, 2, & 4 kHz) for the non-implanted ear at the pre-operative and annual post-activation visits for cochlear implant users with asymmetric hearing loss. The numbers above the panels are the number of participants with available data at each visit. **(A)** Displays the individual results for participants whose PTA did not vary by more than 15 dB HL over the study period (*n* = 29). **(B)** Displays the individual results for the six participants who experienced a decline in the PTA of ≥ 15 dB HL. Symbol shading indicates the individual’s age at surgery, as defined in the legend.

Figure 1 plots the unaided PTA in the non-implanted ear for individual participants at the pre-operative and long-term post-activation visits. Symbol shading indicates the participant’s age at surgery, as defined in the legend. Table 1 lists the LME model results. There was a non-significant main effect of visit (*p* = 0.779), indicating that hearing in the non-implanted ear did not change significantly over the reviewed period. There was a significant main effect of age (*p* < 0.001), with older adults having poorer hearing in the non-implanted ear than younger adults. The interaction between visit and age was non-significant (*p* = 0.114), indicating that the effect of age on the unaided PTA did not change significantly over time.

**Table 1:** Linear mixed model analyzing the main effects and interaction of visit (pre-operative and annual post-activation visits; continuous variable) and age at surgery (years; continuous variable) on the unaided pure-tone average (PTA; 0.5, 1, 2, & 4 kHz; continuous variable) at each visit for adult CI users with asymmetric hearing loss.

Variable	Coefficient	Standard error	df	*t*	*p* value
(Intercept)	−31.10	9.24	197	−3.37	<0.001
Visit	−0.10	0.41	197	−0.26	0.799
Age	0.90	0.15	38	6.04	<0.001
Visit x Age	0.01	0.01	197	1.59	0.114

On the individual-level, the unaided PTA for most participants (*n* = 29; 83%) did not decline by ≥15 dB HL over time (Figure 1A: top row). Six participants (17%) experienced a decline of ≥15 dB HL in the PTA in the non-implanted ear (Figure 1B: bottom row). The case studies for these six participants are reviewed below.

**Figure 2:**
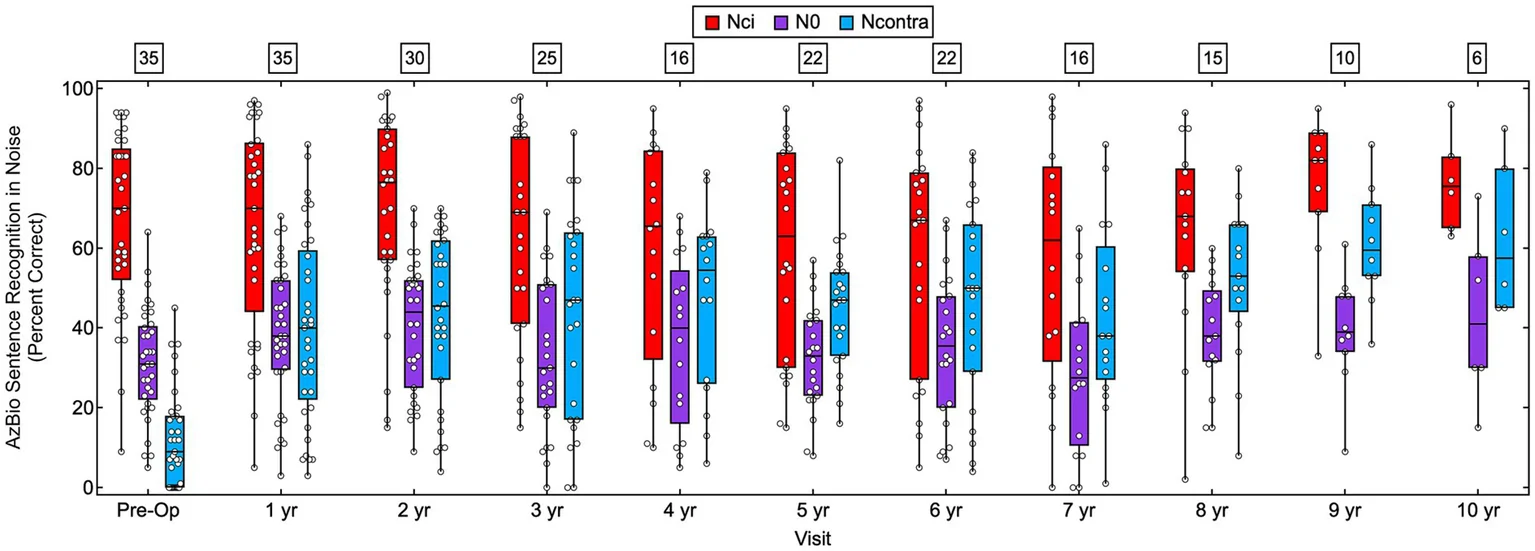
AzBio sentence recognition in noise (10-talker masker) in percent correct for the 3 target-to-masker configurations at the pre-operative and post-activation annual follow-up visits for 35 cochlear implant users with asymmetric hearing loss. The target sentence was presented from the front loudspeaker and the masker was either co-located with the target (N0; purple boxplot), 90° towards the implanted ear (Nci; red boxplot), or 90° towards the contralateral ear (Ncontra; blue boxplot). Individual data are overlaid on the boxplots. The numbers above the figure are the number of participants with available data at each visit.

Figure 2 plots the speech recognition in noise scores (in percent correct) for the 3 target-to-masker configurations (S0Nci, S0N0, and S0Ncontra) at the pre-operative and post-activation visits. Spatial release from masking (SRMci and SRMcontra) can be visualized in Figure 2 as the improvements in speech recognition when the masker was presented towards the implanted ear or contralateral ear as compared to the co-located configuration (S0N0).

Table 2 lists the results for the two LME models that analyzed the main effects of visit (annual post-activation visits), age at surgery, and contralateral PTA on SRMci and SRMcontra. For SRMci, there were no significant main effects of long-term visit (*p* = 0.508) or age at surgery (*p* = 0.067). There was a significant main effect of PTA in the non-implanted ear on SRMci (*p* = 0.002), with more benefit for those with a better PTA. For SRMcontra, there were significant main effects of long-term visit (*p* < 0.001), age at surgery (*p* = 0.002), and PTA in the non-implanted ear (*p* = 0.009). Specifically, greater SRM was experienced over time, by younger participants, and by participants with a poorer PTA.

**Table 2:** Linear mixed model analyzing the main effects of visit (annual post-activation visits; continuous variable), age at surgery (years; continuous variable), and contralateral unaided PTA at each visit (continuous variable) on spatial release from masking (SRM) when the 10-talker masker was presented towards the CI ear (SRMci) or towards the contralateral ear (SRMcontra) for adult CI users with asymmetric hearing loss.

Variable	SRMci					SRMcontra				
	Coefficient	Standard error	df	*t*	*p* value	Coefficient	Standard error	df	*t*	*p* value
(Intercept)	51.04	8.49	115	6.01	<0.001	16.48	9.06	155	1.82	0.071
Visit	0.24	0.37	155	0.66	0.508	1.85	0.38	155	4.93	<0.001
Age	−0.29	0.15	35	−1.89	0.067	−0.54	0.16	35	−3.33	0.002
PTA	−0.33	0.10	155	−3.15	0.002	0.29	0.11	155	2.63	0.009

Interaction terms were not included in the models.

Of interest was whether these results were due in part to most participants with data at the later visits (e.g., 10 years post-activation) being those who had better unaided thresholds in the non-implanted ear. As described above, individuals with single-sided deafness (i.e., normal or near-normal hearing) were enrolled in the clinical trial first; afterwards, recruitment was initiated for individuals with mild to moderate hearing loss in the better ear. Thus, participants with better unaided thresholds in the non-implanted ear have longer term data than those with poorer unaided thresholds. When we limited the data to those obtained between the 1 and 6 years post-activation visits, the pattern of results for the above models did not change – except that there was no longer a significant main effect of PTA for SRMcontra (coef: 0.22, SE: 0.12, t_(108)_ = 1.86, *p* = 0.066). That is, when the data were limited to earlier visits (1–6 years post-activation) to avoid sample bias, SRMcontra was significantly predicted by visit and age, with more benefit over time and for younger adults. Taken together, the finding from the primary model of the significant influence of PTA on SRMcontra should be interpreted with caution, demonstrating the need for longer term data from individuals with poorer PTAs in the non-implanted ear.

### Case studies

**Figure 3:**
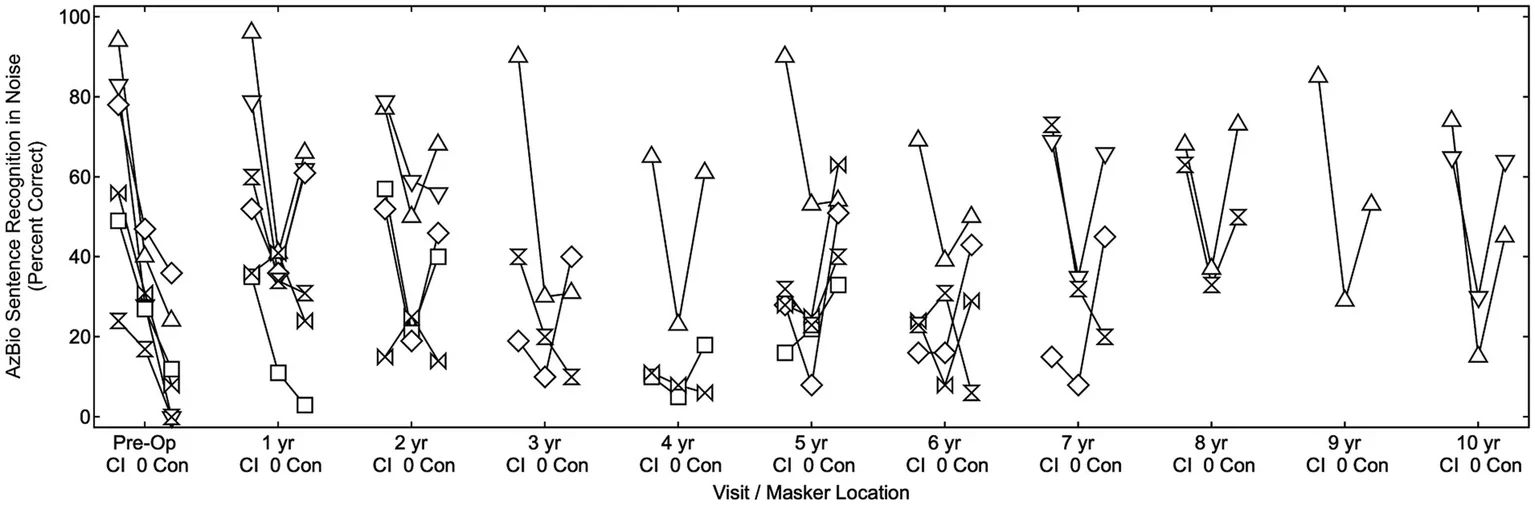
AzBio sentence recognition in noise (10-talker masker) in percent correct for the 3 target-to-masker configurations at the pre-operative and post-activation annual follow-up visits for the 6 participants who experienced a decline in the PTA of ≥ 15 dB HL. The target sentence was presented from the front loudspeaker and the masker was either co-located with the target (0), 90° towards the implanted ear (CI), or 90° towards the contralateral ear (Con).

Of the six participants whose PTA in their non-implanted ear declined by ≥15 dB HL, two were sudden in nature and four were progressive (see Figure 1). Figure 3 plots the speech recognition in noise over time for these individuals. Symbol shape is the same as depicted in Figure 1 to allow for visualization of individual-level results between figures. As expected, individual performance generally worsened with a decline in hearing. For example, one participant (bowtie symbol) experienced a decline in unaided thresholds at 2 years post-activation and demonstrated poorer speech recognition in each target-to-masker configuration compared to their 1 year post-activation visit. This participant experienced fluctuating hearing loss in the non-implanted ear due to recurrent middle ear issues, which influenced their speech recognition in noise over time. Two participants declined from a PTA in the normal hearing range to the mild-to-moderate range: one was fit with a hearing aid (triangle symbol; 8 years post-activation) and one elected not to be fit with a hearing aid (inverted triangle symbol). Another participant (hourglass symbol) experienced a decline in hearing that warranted a change in the acoustic settings for their hearing aid.

For two additional participants (square and diamond symbols), the change in unaided thresholds met the criteria for cochlear implantation; both participants are now bilateral CI users (square at 6 years post-activation and diamond at 8 years post-activation). For the participant represented by the square symbol, performance improved with bilateral CIs, when compared to their bimodal performance at 5 years post activation, in all three target-to-masker configurations. Specifically, scores improved from 16 to 30% for S0Nci, 22 to 35% for S0N0, and 33 to 55% for S0Ncontra. Also, for the participant represented by the diamond symbol, scores improved from 15 to 56% for S0Nci, 8 to 65% for S0N0, and 45 to 68% for S0Ncontra.

## Discussion

Adults with AHL who receive a CI experience significant improvements in speech recognition in noise as compared to pre-operative performance (Dillon et al., 2020; Firszt et al., 2018; Johnson et al., 2025; Thompson et al., 2022), though variability is observed across individuals. The present study investigated the influence of the hearing in the non-implanted ear and age on long-term performance for a group of CI users with AHL who originally received their device as part of a clinical trial. For most participants, the PTA in the non-implanted ear did not change significantly over the reviewed post-activation period. Also, spatial hearing benefits after 1-year post-activation either did not change significantly (SRMci) or continued to improve significantly (SRMcontra) over the reviewed long-term post-activation period. Spatial release from masking was significantly influenced by the hearing in the non-implanted ear when the masker was spatially-separated toward the CI ear – with participants with better unaided thresholds in the non-implanted ear experiencing greater SRM than participants with poorer thresholds. Also, SRM was significantly influenced by age when the masker was spatially-separated towards the non-implanted ear – with younger participants experiencing greater SRM than older participants. Taken together, these data demonstrate that benefits of CI use are maintained with long-term device use and reinforce the need for long-term monitoring of unaided thresholds in the non-implanted ear as it is one of the variables that influences individual outcomes for CI users with AHL.

The unaided thresholds in the non-implanted ear did not significantly change for the majority of the present sample over the reviewed long-term visits. These data are in contrast to those of Mertens et al. (2017) who assessed outcomes at a long-term post-activation visit for 23 CI users with AHL, and found that the unaided hearing in the non-implanted ear was significantly different at the post-activation visit (range: 3–10 years; mean: 7 years) compared to the pre-operative visit. One difference between the samples is that the primary reason for surgery was incapacitating tinnitus for the Mertens et al. sample, whereas for the present sample, enrollment in the original clinical trial was limited to individuals with perceived tinnitus severity of moderate or less. Also, a consideration of the present data is the timeline for when the group with SSD was enrolled versus those with poorer hearing in the non-implanted ear. The original clinical trial first enrolled a group of individuals with SSD (normal to near-normal hearing in the non-implanted ear), and then enrolled a group of individuals with mild to moderate hearing loss in the non-implanted ear. That is, participants in the first group have longer term data than participants in the second group. There is a need for studies with larger sample sizes to understand the variables that influence the long-term stability of hearing for individuals with AHL.

The unaided hearing in the non-implanted ear significantly influenced SRM when the masker was spatially-separated towards the implanted ear (SRMci), demonstrating the importance of this variable in understanding individual outcomes. The significant influence of unaided hearing in the non-implanted ear on outcomes for CI users with AHL has been reported in other samples (Dillon et al., 2026; Firszt et al., 2018; Mertens et al., 2017). For example, Mertens et al. (2017) stratified their sample into those with SSD (PTA ≤ 30 dB HL; *n* = 12) and those with AHL (PTA > 30 dB HL; *n* = 11) and showed that SRMci was greater for the SSD group than for the AHL group at their long-term visit. Similarly, Firszt et al. (2018) showed better speech recognition in noise scores in the bimodal condition for a group of CI users with AHL who had better hearing in the non-implanted ear than for groups with poorer hearing. These combined studies demonstrate that individual outcomes are influenced in part by the hearing in the non-implanted ear. The counseling of CI candidates with AHL should include the influence of the hearing in the non-implanted ear to support realistic expectations of potential individual outcomes.

When the masker was spatially-separated towards the non-implanted ear (SRMcontra), SRM was significantly predicted by the individual’s age at surgery. The magnitude of SRM may be influenced by auditory aging, with greater SRM observed for younger adults than for older adults (Gallun et al., 2013; Srinivasan et al., 2016). For example, Dillon et al. (2020) reviewed the SRM for adult CI users with either SSD (unaided thresholds 125–8,000 Hz ≤ 35 dB HL; *n* = 20) or AHL (pure tone average at 0.5, 1, & 2 kHz between 35 and 55 dB HL, *n* = 15) at 1-year post-activation as a function of age at surgery. They reported that younger adults generally had greater SRM than older adults, but were unable to disentangle the effects of age from the hearing in the non-implanted ear. In a different study that included the effects of age on performance of CI users with AHL, Bernstein et al. (2020) assessed the speech recognition of 13 CI users with AHL (mean age at test: 56 years, 36–74 years) who had a mean of 3 years of CI use (range: 0.7–7 years). Performance was assessed in a monaural and a bilateral condition. For the monaural condition, the target and masker (competing-speech) were presented to the CI ear. In the bilateral condition, the masker (competing-speech) was also presented to the non-implanted ear. Participants experienced a significant interference (poorer performance in the bilateral condition than the monaural condition); the magnitude of this interference was found to be significantly influenced by age, with younger adults experiencing less interference than older adults. Taken together, these data demonstrate that the variability in individual outcomes are influenced in part by aging effects. Importantly, there are data demonstrating that older adults with AHL experience benefits with their CI as compared to their own pre-operative abilities (Dillon et al., 2026; Johnson et al., 2025). Thus, counseling of CI candidates with AHL should include the potential benefits of CI use and the influence of age on an individual’s outcome.

There are limitations of the present study worth consideration. Some participants had ceiling-level performance in the S0Nci target-to-masker configuration (see Figures 2, 3), which limits the calculated SRM when spatially-separating the masker towards the implanted ear. Data at the later visits (e.g., 10 years post-activation) were mostly from individuals with better hearing in the non-implanted ear, which may limit the generalizability of the later visit trends to the broader AHL population. Additionally, the statistical model may have attenuated non-linear changes during the reviewed period. Also, not all participants returned annually and some participants withdrew from the study (e.g., due to moving out of state, other health needs), which limited the available data. It is unclear whether factors related to discontinuing follow-up bias the present results. Additionally, other variables (e.g., cognition, auditory processing) that may be associated with aging and potentially underlying the observed effects were not evaluated in the present study. Also, mapping variables (e.g., procedure to assign MCL levels, filter frequencies) were not controlled in this study. The sample also included participants who were CI-alone users. There are emerging data on the benefits for EAS users with UHL (Dillon et al., 2024; Lorens et al., 2021), however, it is unclear whether the present results would generalize to CI recipients with functional hearing preservation. Finally, SRM for CI users with AHL has been shown to be influenced by the angular separation of the loudspeakers presenting the target and the masker (Anderson et al., 2022), with greater SRM experienced when the loudspeakers are further apart. It is unclear whether the results of the present study would be observed for speech recognition measured with loudspeakers placed closer together than in the present study set-up (e.g., 45° separation versus 90°).

## Conclusion

Most adult CI users with AHL did not experience a significant change in their hearing in the non-implanted ear in the long-term post-activation period (2–10 years post-activation). Unaided hearing in the non-implanted ear and age significantly influenced the benefit experienced by spatially-separating the masker from the target, with unaided hearing influencing SRMci (masker towards the implanted ear) and age influencing SRMcontra (masker towards the non-implanted ear). The role of these variables should be included in the counseling for CI candidates with AHL to establish realistic expectations. These variables should also be included in the analyses of long-term outcomes to understand the individual variability in performance.

## Data Availability

The datasets presented in this article are not readily available because preliminary data are part of an ongoing investigation.
